# Climate change and infectious diseases: translating evidence into action

**DOI:** 10.1186/s40249-026-01468-z

**Published:** 2026-06-10

**Authors:** Xiaoming Shi, Tiantian Li, Yu Wang, Qiyong Liu, Jiao Wang, Jie Ban, Patrick L. Kinney, Shilu Tong

**Affiliations:** 1 National Key Laboratory of Intelligent Tracking and Forecasting for Infectious Diseases, National Institute of Environmental Health, Chinese Center for Disease Control and Prevention, Beijing, 100021 China; 2 China CDC Key Laboratory of Environment and Human Health, National Institute of Environmental Health, Chinese Center for Disease Control and Prevention, Beijing, 100021 China; 3 National Key Laboratory of Intelligent Tracking and Forecasting for Infectious Diseases, National Institute for Communicable Disease Control and Prevention, Chinese Center for Disease Control and Prevention, Beijing, 102206 China; 4 Department of Environmental Health, Boston University School of Public Health, Boston, MA USA; 5 School of Public Health and Social Work, Queensland University of Technology, Brisbane, QLD Australia

**Keywords:** Climate change, Infectious disease, Health impact, Response strategy, Action

## Abstract

Climate change is reshaping the global landscape and transmission of infectious diseases, posing a profound threat to public health systems. Despite a growing body of evidence on expanding transmission of infectious diseases due to climate change, translating this evidence into effective actions to reduce infectious disease burden remains insufficient. To address these challenges, we organized this special issue *Climate Change and Infectious Diseases* and synthesized new evidence on climate-sensitive infectious diseases and response frameworks. To bridge the implementation gap, we propose a core framework of four evidence-based strategies to mitigate impacts of climate change on infectious diseases, including strengthening health adaptation actions, applying innovative and comprehensive control measures, building climate-resilient and low-carbon health systems, and improving global governance and equity. We advocate for translating evidence into action to reduce infectious disease burdens in the context of global climate change, and policymakers, researchers, and clinical practitioners need to work together to achieve this important goal.

## Background

Climate change is the biggest global health threat of the twenty-first century [1], acting as a risk multiplier that amplifies the landscape and transmission of vector-borne, foodborne, waterborne, and airborne infectious diseases [2]. Rising temperatures, irregular rainfalls and more frequent and intense extreme weather events have far-reaching impacts on human health, environment, animals, and food systems [3, 4]. These shifts influence the geographic distribution, transmission efficiency, and seasonality of infectious diseases by altering pathogen dynamics, vector and host behavior, and human–environment interactions [3]. Climatic hazards such as warming, precipitation and floods are likely to heighten the severity of 58% (218 out of 375) pathogens and over 1000 transmission pathways of infectious diseases [5]. Annual global dengue infections are expected to increase to 77 (confidence interval: 40, 198) million during 2041–2060 under the Shared Socio-economic Pathway 2–4.5 compared with 49 (26, 127) million in 2000–2019, with significant expansion in higher latitudes such as North America and Europe [6]. Notably, the escalating risk and burden of infectious diseases are not equitably distributed. The low- and middle-income countries (LMICs) and tropical regions face disproportionate higher threats, due to weaker health systems, limited adaptive capacity, and heightened exposure to climate hazards [4].

However, mitigating these risks in a changing climate remains a formidable challenge. Despite growing recognition, several scientific and technical challenges impede effective responses to the rising burdens of infectious diseases. For example, local and real-time surveillance and early warning systems for diseases are necessary to detect the early signals of disease outbreaks but are often constrained by inter-ministerial data silos and a lack of operational mechanisms for multisectoral data sharing [7]. Community-based and cost-effective disaster risk management (e.g., individual-level health education and community-based One Health approach intervention programs) remains largely theoretical [8]. To address these challenges, we organized this special issue *Climate Change and Infectious Diseases* and included ten studies. Drawing on these research advances and emerging global evidence, we aim to synthesize fresh insights into climate-sensitive infectious diseases across diverse geographical regions and to provide policymakers, researchers, and healthcare practitioners with a global perspective, thereby advancing the translation of evidence into action against the growing burden of infectious diseases.

## Evidence on climate-sensitive infectious diseases and global response strategies

Abnormal meteorological conditions and extreme weather events catalyzed by climate change are amplifying the risks and facilitating the range expansion and geographic redistribution of infectious diseases [9]. Temperature, precipitation, and humidity are major climate drivers influencing the emergence, replication and distribution of pathogens, including the dengue virus, West Nile virus, influenza virus and *cryptosporidium* [9–11]. Concurrently, these climate factors also affect the survival, fecundity, and transmission-related behavior of vectors (e.g., *Aedes albopictus* and ticks) [12, 13] and hosts (e.g., small mammals, birds, and *Bulinus globosus*) [9, 14].

Evidence suggests that climate change drives the expansion of infectious diseases into historically temperate climates or unsuitable regions. An accelerating northward trend of *Aedes albopictus* and corresponding expansion of dengue and chikungunya transmission risks have been observed in many parts of the world, including China and Europe, and the dengue risk is projected to expand to nearly all of China by the 2050s [12, 15]. Beyond the expansion of transmission risk, climate change promotes a dynamic shift among infectious diseases. Warming temperatures in Africa may favor the transmission of *Aedes aegypti*-borne diseases (e.g., dengue, chikungunya, and Zika) over malaria, due to a higher thermal performance of *Aedes aegypti* compared with *Anopheles gambiae* [16]. Furthermore, the impact of climate change varies significantly by vector’s life spans, with extreme weather events shown to affect population sizes of short-lived mosquitoes more than those of long-lived ticks [9]. However, it is important to note that, at the backdrop of climate warming, tick-borne infections (e.g., Alongshan virus, Beiji nairovirus, and rickettsiosis) have been increasingly emerging and expanding in China, with 28 types of tick-borne infectious diseases reported during 2015–2025, nearly double the 15 types in 1980–2015 [17].

Climate change alters the transmission efficiency and seasonality of infectious diseases through complex and sometimes unpredictable biological interactions. Temperatures and other climate factors can influence the vector competence (i.e., intrinsic ability of transmission) and vectorial capacity (e.g., extrinsic incubation period) [9]. High temperatures may increase vector competence, but reduce survival of adult female mosquitos. Temperatures can affect both viral replication and ribonucleic acid interference (RNAi) suppressing viral replication in vectors [9]. These biological interactions and balances ultimately determine the infection risks and seasonality of infectious diseases. For instance, the dominant seasonal patterns of influenza in Africa do not always align with classical northern hemisphere or southern hemisphere patterns, which may be more applicable in temperate climates [18]. In China, the arid, desert, and cold climates experience the earliest peak of influenza annually, whereas only tropical and specific temperate climates show summer peaks [19]. Understanding these climate- and regional-specific epidemic periods may facilitate developing targeted early warning systems, optimizing vaccination timing, and implementing target interventions.

In the era of climate change, global strategies for preventing pandemics of emerging infectious diseases advocate for One Health approach and integrated actions from multisectors [20]. One Health approach aims to sustainably balance and optimize the health of people, animals, and ecosystems [21]. Through interconnected actions among human, animal, and environment, it can enhance both primary prevention (blocking viral spillovers) and secondary prevention (curbing spread among humans after viral spillovers) for viral pandemics [22, 23]. For example in Pemba, Tanzania, a One Health approach comprising dog vaccination and emergency human post-exposure vaccination was adopted to control dog-mediated rabies, leading to the elimination of human rabies cases from 2010 to 2014 [24]. It is also critical to move beyond crisis response to integrated actions to cope with the impacts of climate change. For example, Cape Town, South Africa, implemented multisectoral health-related interventions to prevent diarrhea disease, with a sustained health response to climate variability (e.g., common community-level health education, emergency rehydration services, and environmental and lifestyle etiology assessment for diarrhoeal cases), resulting in a 64.7% reduction in diarrhea incidence, with benefits persisting even during severe droughts [25]. However, disproportionately less scientific attention and endemic control measures have been directed towards risks of non-vector-borne infectious diseases and in tropical regions and LMICs, reflecting existing gaps, inequities and key directions for advancing infectious disease control [26, 27].

## Translating evidence into action

**Fig. 1:**
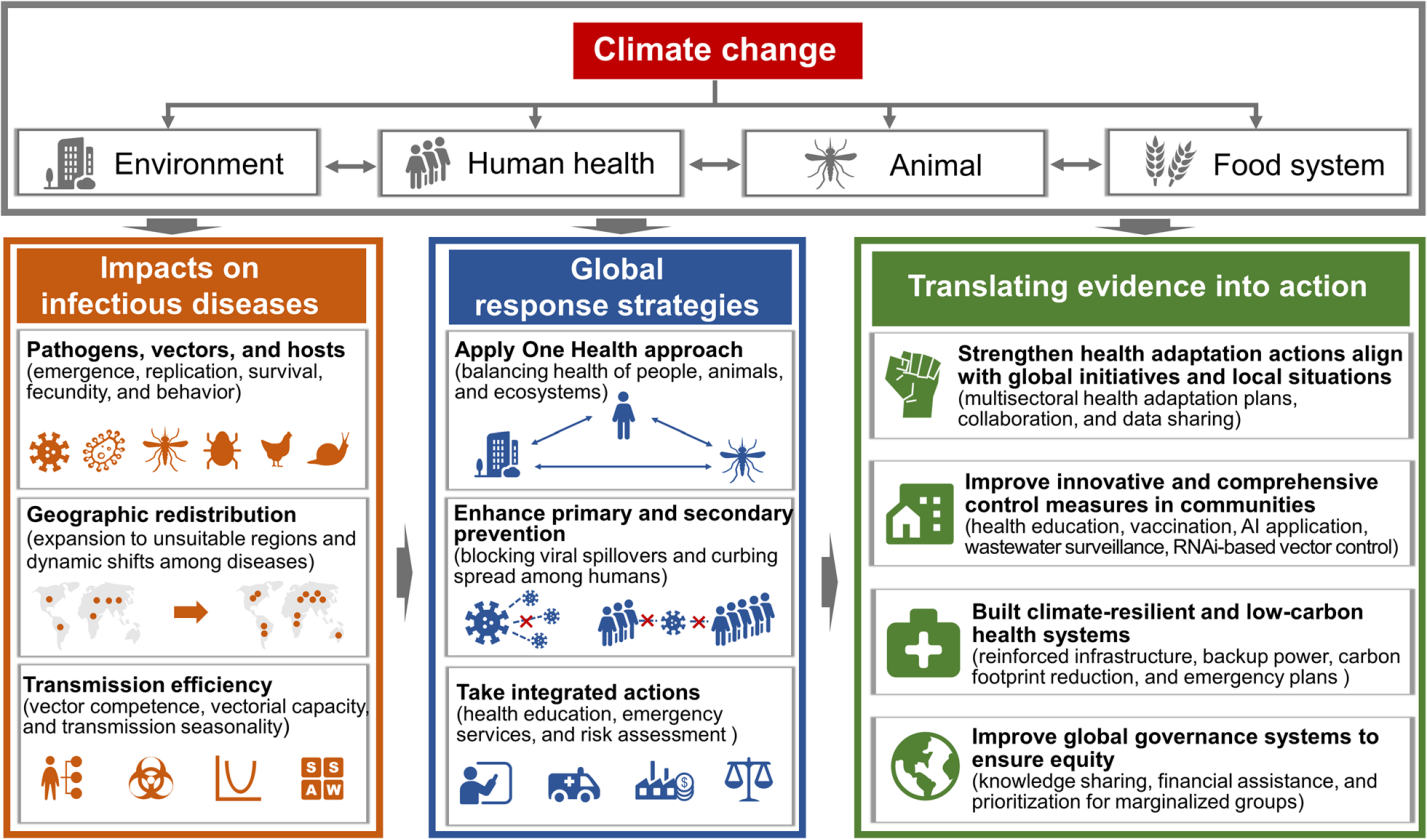
Graph summary of the impacts of climate change on infectious diseases and evidence-based strategies

The 2025 World Health Assembly acknowledges the profound impact of climate change on infectious diseases and underscores the urgency for global action [28]. Both the 29th and 30th Conferences of the Parties highlight the importance of integrating health issues into climate action, advocating for climate-informed global health surveillance for infectious diseases and One Health approach for zoonotic disease control [29, 30]. Based on these calls, countries like England have implemented weather and health warnings, while China has issued national climate change health adaptation actions [31]*.* Despite these commitments, translation into effective action faces significant systemic barriers. These include fragmented sectoral governance that hinders multisectoral coordination for climate change and health [20], a lack of locally technical guidance for implementation [32], an unstable global political and policy environment [33], and inadequate technical and financial support for LMICs [27]. Here, we propose four key evidence-based strategies to mitigate the impacts of climate change on infectious diseases (Fig. 1).

### Health adaptation actions align with global initiatives and local situations need to be strengthened

The World Health Organization and other global organizations emphasize the necessity to place health at the centre of national adaptation plans, and have already outlined criteria and high-value actions for health sector adaptation, such as climate-informed early warning systems and community-level health education programs [7, 32]. Releasing health adaptation plans is the fundamental action to ensure health leadership and promote multisectoral endorsement of climate and health interventions [32]. However, effective implementation of adaptation actions requires coordinated efforts from governments at all levels, relevant sectors and stakeholder partnerships, and integrating open science on climate, ecosystem, sociodemographic features, and infectious diseases into core health policies [34]. This necessitates improving collaboration and data sharing across health, meteorology, environmental, agricultural, water, urban planning, and disaster management sectors. Notably, actions must be evidence-based and locally targeted, with priority task plans developed for identified high-risk areas and vulnerable populations [31]. Public awareness and education campaigns should be integrated into adaptation plans in communities, including community-based workshops, digital information platforms, and school-based health education programs [35].

### Innovative and comprehensive control measures are needed to improve the community resilience against infectious diseases

Under the framework of local health adaptation action plans, combination of traditional measures (e.g., health education, preventive drugs, and vaccination) and novel techniques (e.g., wastewater surveillance, *Wolbachia*-infected or RNAi-based mosquito vector control, and new vaccines for dengue) [36–38] are needed to improve the community resilience against the changing patterns of infectious disease. Artificial intelligence (AI) has been applied to combat pandemic through all outbreak stages, including disease and pathogen monitoring, transmission risk assessment and early warning detection, disease diagnosis and prognostic prediction, and vaccine design [39]. They can enable intelligent, precise, and efficient health warnings and decision makings derived from multisectoral, complex, and heterogeneous data [40]. However, some advanced technologies require digital, technical, and financial capacity, which is often lacking in most LMICs. Therefore, frugal and context-appropriate strategies—such as community-level health education, locally-sourced vector control, utilizing mobile phones for real-time case reporting and risk assessment, and medical supply backup—are more likely to be implemented and sustained.

### Climate-resilient and low-carbon health systems need to be built to address the urgency

It is essential not only to enhance the overall adaptive capacity of the health systems (e.g., public health agencies, community health centers, and hospitals) but also to directly ensure the continuity and effectiveness of infectious disease prevention and control efforts. Health systems must maintain critical functions during extreme weather events particularly in LMICs. Reinforcing infrastructure against flooding, securing backup power, water, and medical supplies can guarantee uninterrupted disease surveillance, laboratory testing, and medical care [41]. In areas with financial and technical capacity, mitigating health sector’s carbon footprint is vital to reduce environmental pollution and potential impacts on disease transmission. Key actions include adopting green building standards, implementing smart energy management to minimize waste, and promoting zero-emission transportation [42]. Furthermore, it is necessary to develop and refine both routine and emergency plans, including infectious disease contingency plans, to ensure the continuity of core services during various public health emergencies, thereby effectively safeguarding population health.

### Global governance systems must be improved to ensure the realization of equity

Systematically integrating equity principles into governance frameworks is required and these principles should be implemented through the whole decision-making process. Essential actions include strengthening knowledge sharing, optimizing the allocation of resources for infectious disease prevention and control, improving mechanisms for financial and technical assistance for LMICs, and establishing coordinated systems for monitoring cross-border threats, including emerging infectious diseases [41, 43]. In this process, governance must prioritize marginalized groups — such as children, the elderly, impoverished communities, and displaced persons — and vulnerable regions, including LMICs, small island developing states, and conflict-affected areas. These groups and regions have contributed the least to climate change yet bear the most climate impacts, including an increased burden of infectious diseases. Global governance mechanisms must therefore focus on correcting this imbalance, and move the whole society forward.

## Conclusions

Climate change reshapes the transmission and landscape of infectious diseases, threatening global public health security and equity. This comment synthesizes evidence and proposes four interconnected evidence-based strategies to address barriers in translating evidence into effective action. We must move beyond rhetoric toward integrated and operational implementation that reduces infectious disease burdens in the context of global climate change.

## Data Availability

All the materials used in this manuscript can be accessed through the DOI or website listed in references.

## Abbreviations

LMICs: Low- and middle-income countries
AI: Artificial intelligence
RNAi: Ribonucleic acid interference
